# Mesenteric desmoid tumor with diagnostic challenge mimicking a malignant small intestinal tumor: a case report

**DOI:** 10.1097/RC9.0000000000000533

**Published:** 2026-05-21

**Authors:** Teppei Tokumaru, Motoyasu Tabuchi, Sunao Uemura, Shuta Tamura, Jun Iwata, Takehiro Okabayashi

**Affiliations:** a Department of Gastroenterological Surgery, Kochi Health Sciences Center, Kochi, Japan; b Department of Pathology, Kochi Health Sciences Center, Kochi, Japan

**Keywords:** case report, diagnostic challenge, intra-abdominal tumor, mesenteric desmoid tumor, surgical resection

## Abstract

**Introduction and importance:**

Desmoid tumors are histologically benign fibroblastic neoplasms with locally aggressive behavior and no metastatic potential. Intra-abdominal desmoid tumors often lack specific imaging features, and preoperative tissue diagnosis may be difficult, complicating treatment decision-making.

**Case presentation:**

A 66-year-old man presented with a painless abdominal mass sensation. Contrast-enhanced computed tomography revealed a well-circumscribed intra-abdominal mass measuring approximately 12 × 10 cm, adjacent to the small intestine, with heterogeneous enhancement and internal low-attenuation areas, raising suspicion of a malignant small intestinal tumor, particularly a gastrointestinal stromal tumor. Surgical resection was performed without a preoperative biopsy due to persistent symptoms and diagnostic uncertainty. The tumor was located within the small bowel mesentery and was resected en bloc with the adjacent small intestine. Histopathological examination confirmed a mesenteric desmoid tumor. Familial adenomatous polyposis was absent, and the tumor was considered sporadic.

**Clinical discussion:**

Mesenteric desmoid tumors may cause tumor-related symptoms without bowel obstruction or overt invasion, and their nonspecific imaging characteristics may lead to diagnostic challenges. In such situations, diagnostic uncertainty can directly influence surgical decision-making and treatment strategy.

**Conclusion:**

This case highlights the importance of considering desmoid tumors in the differential diagnosis of intra-abdominal masses and demonstrates how diagnostic uncertainty can influence the treatment strategy.

## Introduction

Desmoid tumors are histologically benign fibroblastic neoplasms characterized by locally aggressive growth without metastatic potential^[^1–3^]^. Their biological behavior varies greatly, ranging from spontaneous stabilization or regression to progressive local growth, causing significant morbidity^[^1–3^]^. Active surveillance is an accepted management strategy following a definitive diagnosis^[^1–4^]^. Desmoid tumors are classified into sporadic (85–90%) and familial adenomatous polyposis (FAP)-associated types (10–15%)^[^1,2^]^.

Intra-abdominal desmoid tumors often lack specific imaging features and may closely resemble other mesenchymal or malignant tumors^[^1,3,5^]^. Furthermore, preoperative tissue diagnosis is frequently challenging because biopsy of intra-abdominal tumors may be limited by anatomical constraints and concerns regarding tumor dissemination or injury to adjacent organs when malignancy cannot be excluded^[^1,4^]^. Consequently, treatment decisions may be complicated by diagnostic uncertainty.

HIGHLIGHTS

- Mesenteric desmoid tumors may closely mimic malignant small intestinal tumors in imaging studies.
- Definitive preoperative diagnosis is often difficult because the biopsy of intra-abdominal tumors is limited.
- Diagnostic uncertainty can necessitate surgical intervention despite the benign nature of desmoid tumors.
- Surgical decision-making should prioritize oncological safety when malignancy cannot be confidently excluded.

Herein, we report a case of a mesenteric desmoid tumor that could not be diagnosed preoperatively and required surgical decision-making under diagnostic uncertainty. Diagnostic uncertainty directly influenced the decision to proceed with surgical resection without preoperative histological confirmation. This case report has been documented in line with the SCARE checklist^[^6^]^.

## Case presentation

**Figure 1.**
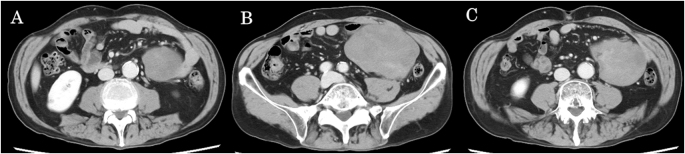
(A) Contrast-enhanced computed tomography images show an intra-abdominal mass adjacent to the ileum. (B, C) The mass was a large, well-circumscribed solid lesion measuring approximately 12 × 10 cm, with mild-to-moderate heterogeneous enhancement.

A 66-year-old man with no significant medical history presented with a 1-month history of a painless, palpable abdominal mass. He denied weight loss, fever, anorexia, nausea, or changes in bowel habits. He had no remarkable family history, prior abdominal surgery, or abdominal trauma. At initial presentation, abdominal examination revealed a palpable, non-tender mass in the lower abdomen. Subsequent contrast-enhanced computed tomography revealed a large, well-circumscribed solid mass measuring 12 × 10 cm, with mild-to-moderate heterogeneous enhancement and an area of relatively low attenuation. The mass displaced adjacent bowel loops without obstruction or ischemia, and no distant disease was detected. On imaging, the mass was found to be closely associated with the ileum, appearing as if originating from the small intestine. Based on these imaging findings, a malignant tumor of small intestinal origin, specifically a gastrointestinal stromal tumor (GIST), was initially suspected (Fig. 1A–C).

Two weeks after the initial CT examination, given the large tumor size, the presence of symptoms, and diagnostic uncertainty, surgical resection was performed without a preoperative biopsy because of concerns regarding potential bowel injury and tumor seeding in the case of malignancy.

**Figure 2.**
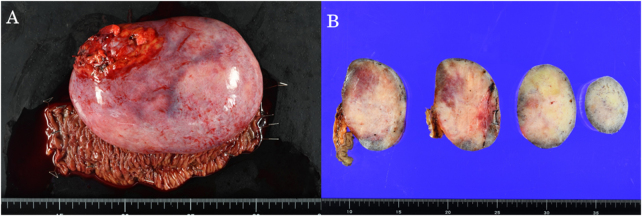
(A, B) Macroscopic appearance of the resected specimen shows a well-circumscribed solid mass with a firm, whitish cut surface. The tumor exhibits a homogeneous fibrous appearance without apparent hemorrhage or necrosis.

Laparotomy showed the tumor's location within the small bowel mesentery, with no evidence of invasion into the small intestine or other adjacent organs. Since malignancy could not be ruled out, the tumor was resected en bloc with the adjacent segment of small bowel supplied by the involved mesenteric vessels, followed by primary anastomosis. Gross examination of the resected specimen revealed a solid mesenteric mass measuring 15 cm × 11 cm × 7 cm. Macroscopic findings suggested a well-circumscribed solid mass with a firm, whitish cut surface. No obvious hemorrhage or necrosis was observed (Fig. 2A and B).

**Figure 3.**
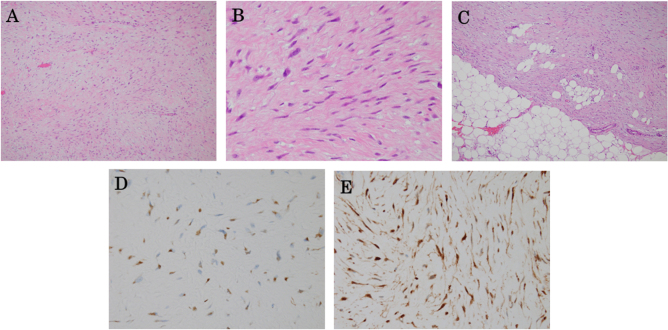
(A) Hematoxylin and eosin staining shows spindle-shaped tumor cells arranged in fascicles (original magnification ×100). (B) Higher-power view demonstrates bland spindle-shaped cells with minimal cytological atypia (original magnification ×400). (C) Focal infiltrative growth of the tumor at the interface with the surrounding adipose tissue (original magnification ×400). (D) Immunohistochemical staining for α-SMA shows partial positivity (original magnification ×400). (E) Immunohistochemical staining for β-catenin shows nuclear accumulation in tumor cells (original magnification ×400).

Histological examination revealed a sparse proliferation of spindle-shaped and stellate fibroblastic/myofibroblastic cells within a fibrous stroma. Some tumor cells showed mild nuclear enlargement, but no obvious nuclear atypia was noted. Keloid-like collagen fibers were observed in some areas. Although the tumor appeared well-circumscribed, mild infiltrative growth into the adjacent adipose tissue was revealed at the periphery. Immunohistochemical staining revealed nuclear accumulation of β-catenin (Fig. 3A–E); c-KIT and DOG1 were negative. These findings confirmed the diagnosis of a mesenteric desmoid tumor. Total colonoscopy was performed to evaluate the possibility of an FAP-associated desmoid tumor, revealing no abnormal findings. Given the patient’s age and the absence of a family history, genetic testing was not performed, and the tumor was considered a non-FAP desmoid tumor. The postoperative course was uneventful, and the patient was discharged on postoperative day 9. At the 3-month follow-up, no evidence of recurrence was observed on CT. The patient will continue to undergo imaging surveillance, considering the potential risk of local recurrence.

## Discussion

This case highlights how diagnostic uncertainty in intra-abdominal desmoid tumors can directly influence treatment strategy. Desmoid tumors exhibit heterogeneous biological behavior and variable imaging findings, and definitive diagnosis is particularly challenging in intra-abdominal lesions^[^1–3,5^]^.

Mesenteric desmoid tumors may demonstrate expansile growth without obvious invasion of adjacent organs or bowel obstruction, even when the tumor is large^[^1–3^]^. Although desmoid tumors are histologically classified as benign fibrous neoplasms, their local growth patterns and clinical presentations vary depending on the anatomical location and biological behavior^[^1–3^]^. In intra-abdominal cases, some tumors remain asymptomatic for prolonged periods and become clinically apparent only after enlargement due to mass effect, whereas others exhibit infiltrative growth involving the bowel or surrounding organs, resulting in intestinal obstruction or ischemia^[^1–3^]^. Sporadic desmoid tumors not associated with FAP (non-FAP) have been reported to show a relatively less aggressive clinical course compared with FAP-associated tumors; however, tumor behavior in non-FAP cases is not uniform, and no definitive predictors of biological aggressiveness have been established^[^1,2^]^. Here, the mesenteric desmoid tumor exhibited expansile growth without obvious invasion of adjacent organs or bowel obstruction and was detected based solely on a painless abdominal mass sensation. Despite its large size, no other clinical symptoms or complications were observed. Histopathological examination revealed focal infiltrative growth at the interface with the adjacent mesenteric adipose tissue, indicating that this tumor exhibited both expansile and infiltrative growth patterns.

Contrast-enhanced computed tomography revealed a well-circumscribed mass adjacent to the small intestine, with relatively low overall enhancement and internal low attenuation. These imaging features can be observed in large GISTs with intratumoral degeneration, hemorrhage, or necrosis; therefore, malignancy could not be ruled out based on imaging findings alone^[^1,3,5^]^. Although histopathological evaluation is essential for a definitive diagnosis, percutaneous biopsy of intra-abdominal tumors is often limited when malignancy cannot be confidently ruled out because of concerns regarding tumor dissemination and potential injury to adjacent organs^[^4,7^]^. Positron emission tomography–computed tomography was considered but not performed, as it is primarily useful for detecting metastasis or recurrence and has limited value for qualitative differentiation^[^1^]^. Furthermore, it was unlikely to alter the treatment strategy in this case.

Endoscopic ultrasound–guided fine-needle aspiration may be considered depending on tumor location; however, its diagnostic yield for desmoid tumors is inconsistent, and a conclusive preoperative diagnosis is not always achievable^[^1,3,5^]^. A discrepancy was noted between the tumor size on preoperative imaging (approximately 12 cm) and that in the resected specimen (approximately 15 cm), which may reflect interval tumor growth and limitations in radiological assessment. Focal infiltrative growth into the surrounding adipose tissue, as observed histologically, may have also contributed to this discrepancy.

Although active surveillance is an established management option once a diagnosis of desmoid tumor has been confirmed, its application is difficult in cases of diagnostic uncertainty^[^1,2^]^. Magnetic resonance imaging (MRI) is useful for evaluating internal tumor characteristics and biological activity in desmoid tumors; however, its reliability in distinguishing intra-abdominal desmoid tumors from other mesenchymal tumors is limited^[^1,5^]^. Here, an MRI was not performed as it was unlikely to provide additional information that would substantially alter the treatment strategy. This case highlights the difficulty of treatment decision-making when a definitive preoperative diagnosis cannot be established.

Desmoid tumors are associated with FAP, and their evaluation is clinically important^[^8,9^]^. In this case, the likelihood of FAP was considered low, given the patient’s age and the absence of a relevant family history. Accordingly, FAP was clinically assessed by total colonoscopy without genetic testing^[^8^]^, which was considered a practical and appropriate approach in routine clinical practice. A definitive preoperative diagnosis was not established, reflecting the diagnostic uncertainty inherent in this case. The follow-up period was short, and continued surveillance is warranted.

## Conclusion

This case illustrates that mesenteric desmoid tumors can grow predominantly in an expansile manner, with focal infiltrative growth and without bowel obstruction or overt invasion, even when the tumor is large. Nonspecific imaging findings and the limited feasibility of tissue biopsy in intra-abdominal tumors may lead to diagnostic uncertainty, which can directly influence treatment strategy. Further accumulation of similar cases may contribute to improved prediction of tumor behavior and refinement of diagnostic strategies for mesenteric desmoid tumors.

## Data Availability

All relevant data supporting the findings of this case report are included in this article. Additional information is available from the corresponding author upon reasonable request.
